# Posture-induced modulation of lower-limb joint powers in perturbed running

**DOI:** 10.1371/journal.pone.0302867

**Published:** 2024-05-14

**Authors:** Soran AminiAghdam, Christian Rode

**Affiliations:** 1 Carnegie School of Sport, Leeds Beckett University, Leeds, United Kingdom; 2 Department of Motion Science, Institute of Sport Science, Friedrich-Schiller-University, Jena, Germany; 3 Department of Biomechanics, Institute of Sport Science, University of Rostock, Rostock, Germany; Università degli Studi di Milano: Universita degli Studi di Milano, ITALY

## Abstract

Despite evidence on trunk flexion’s impact on locomotion mechanics, its role in modulating lower-limb energetics during perturbed running remains underexplored. Therefore, we investigated posture-induced power redistribution in the lower-limb joints (hip, knee, and ankle), along with the relative contribution from each joint to total lower-limb average positive and negative mechanical powers (i.e., over time) during perturbed running. Twelve runners (50% female) ran at self-selected (~15°) and three more sagittal trunk inclinations (backward, ~0°; low forward, ~20°; high forward, ~25°) on a custom-built runway, incorporating both a level surface and a 10 cm visible drop-step positioned midway, while simultaneously recording three-dimensional kinematics and kinetics. We used inverse dynamics analysis to determine moments and powers in lower-limb joints. Increasing the trunk forward inclination yielded the following changes in lower-limb mechanics: a) an elevation in total positive power with a distoproximal shift and a reduction in total negative power; b) systematic increases in hip positive power, coupled with decreased and increased contribution to total negative (during level-step) and positive (during drop-step) powers, respectively; c) reductions in both negative and positive knee powers, along with a decrease in its contribution to total positive power. Regardless of the trunk posture, accommodating drop-steps while running demands elevated total limb negative and positive powers with the ankle as a primary source of energy absorption and generation. Leaning the trunk more forward induces a distoproximal shift in positive power, whereas leaning backward exerts an opposing influence on negative power within the lower-limb joints.

## Introduction

Outdoor running surfaces naturally exhibit variations in terrain properties such as compliance, gradient, and evenness, necessitating runners to adjust their global and local gait mechanics. Understanding these adaptations is crucial for optimizing running performance and designing lower-limb wearable devices while managing injuries. Despite extensive research on lower-limb biomechanics during steady-state locomotion, the role of the upper-body in perturbed locomotion remains understudied. Given that the upper-body (head, arms, and trunk) accounts for approximately 68% of the body’s total mass [1], even minor adjustments in trunk orientation can significantly impact lower-limb mechanics [2–5].

Preferred sagittal trunk inclination varies significantly among participants [3, 6] and is influenced by factors such as speed of locomotion [7], age [8, 9] or spinal deformities [10]. While uneven running is seemingly associated with modified biomechanics in the lower-limb joints (hip, knee, and ankle) [11, 12], there is limited comprehension of their contribution to mechanical power and work demands in relation to trunk inclination when accommodating perturbations. Previous research suggests that the lower-limb joints adapt to specific gait patterns to meet mechanical demands. For instance, incline walking requires greater positive joint work from the ankle, while decline walking demands greater negative joint work from the knee [13]. However, the relative contribution of each lower-limb joint to total limb positive power appears consistent across different level-ground walking and running speeds [14]. Moreover, the walk–run transition is associated with a shift in power generation from the hip to the ankle [14]. Analyzing each joint’s contribution to total lower-limb power during perturbed running would complement the existing understanding of joint-level mechanics in both steady and unsteady locomotion on level or varied slope ground.

Previous research on perturbed running highlights the resilience of human locomotion to diverse terrain irregularities. Anticipated step-down perturbations induce runners to lower their center of mass and reduce leg stiffness in the preceding contacts, likely through feedforward control strategies, followed by increased leg stiffness and touchdown angle in the perturbed contact [15–17]. In contrast, unanticipated substrate height changes prompt compensatory adjustments in a feedback control fashion within a single step [18]. Moreover, the relative joint contribution to total energy absorption appears to vary with drop height during unanticipated progressive drops in hopping [19], with an increased ankle contribution for drop heights of less than or equal to 10 cm, while higher drop heights resulted in greater contributions from the knee and hip joints. However, the influence of trunk orientation on these dynamics has been largely overlooked [20].

While data on the posture-induced redistribution of power in lower-limb joints in response to surface perturbations is limited, existing research indicates that trunk orientation influences the biomechanical function of the leg during step-down perturbations [21–23]. Our recent investigations into the global and local mechanics of uneven running, particularly focusing on trunk orientation [2, 11], suggest that human runners adjust leg stiffness in response to postural changes and varying ground surfaces. Running with a backward trunk lean leads to a greater touchdown leg angle, increased leg compression, a higher knee flexion angle, and a different moment profile compared to habitual running. This, in turn, results in a more compliant spring-like leg. Notably, in the step-down between level surfaces, leg length, angle, and force all increase regardless of trunk inclination to mitigate the vertical drop of the center of mass [2].

Joint power quantifies the rate of joint work, offering insights into mechanical energy absorption and generation phases. During fast movements such as running, rapid musculoskeletal responses to mechanical perturbations are crucial for maintaining an upright posture and effectively managing energy to sustain or regain stability after such perturbations [19]. Investigating lower-limb joint power provides insights into their role in stability control strategies in unstable or unfamiliar environments. This study aimed to characterize posture-induced power redistribution in lower-limb joints, examining each joint’s relative contribution to total limb average negative and positive powers during perturbed running. We hypothesized that (i) the perturbed limb would exhibit increased total negative and positive powers (average) when stepping into a hole (drop-step) and (ii) decreasing total negative power and increasing total positive power with increasing forward trunk inclination. Additionally, (iii) the perturbed limb would demonstrate a proximodistal shift in joint contribution to total negative power with a backward trunk inclination and the opposite shift in total positive power with increasing trunk flexion angle.

## Materials and methods

### Participants

Twelve (half females) recreational runners (mean ± SD; age: 28.5 ± 5.7 years; body mass index: 2.4 ± 1.9 kg m^-2^; running distance: 15.6 ± 5.3 km week^-1^) with the experience of running on uneven surfaces, free of any current/previous lower-limb surgery/injury or low back pain for at least the last six months voluntarily participated in the study. A minimum sample size of eleven participants was determined from a priori power analysis using G*Power (Version 3.1, University of Dusseldorf, Germany), implementing an effect size of 0.33 and statistical power of 80% (α = 0.05). The study was approved by the local Ethics Committee of the Friedrich-Schiller-University Jena (3532–08/12) and was performed according to the Declaration of Helsinki. All participants were informed of the experimentation’s benefits and risks prior to signing the approved, written consent document to participate in the study scheduled from November 15^th^ to December 15^th^, 2017.

### Experimental design and protocol

Data were collected at the Institute of Sports Science, Friedrich-Schiller-University Jena, utilizing a twelve-camera motion-capture system (250 Hz; MCU1000, Qualisys, Sweden) and two consecutive force plates [1000 Hz; 9281B (0.4 × 0.6 m), 9287BA (0.6 × 0.9 m), Kistler, Switzerland], embedded halfway along a 15 m instrumented track. The arrangement of the force plates allowed for step lengths ranging from 1.40 to 2.30 m. We synchronized kinematics and ground reaction force data using an external trigger and BioWare data acquisition software (Kistler Instrument AG, Switzerland). Applying joint coordinate standards of the International Society of Biomechanics [24], a twelve-body segment model was defined using nineteen reflective markers. The markers were placed bilaterally on the following bony landmarks: fifth metatarsal heads, lateral malleolus, lateral epicondyles of femurs, greater trochanters, anterior superior iliac spines, L5–S1 junction (L5), lateral humeral epicondyles, wrists, acromioclavicular joints, seventh cervical spinous process (C7) and middle of the forehead. The trunk angle was the angle sustained by the line connecting L5 and C7 markers with respect to the vertical [21]. The mean trunk angle was calculated as the average sagittal plane trunk posture during the stance phase of the level step. Participants first ran using their self-selected trunk inclination (*TI*_*0*_). For the low forward (*TI*^+^) and backward (*TI¯*) conditions, participants were instructed to increase and decrease their trunk flexion angle, respectively, to their comfort while running on even or uneven tracks [2, 11]. Additionally, for the high forward (*TI*^++^) trunk inclination, participants had their trunk angle visually compared to a cardboard template by a second examiner before the trials. The adjustable-height template was drawn at a 30° angle and mounted on a wall parallel to the runway. For more details, please see [21].

After completing the run on a level, even track (level-step), the variable-height force plate located at the site of the second contact (drop-step) was lowered by 10 cm. Subsequently, participants ran along the uneven track. The order of the *TI¯*, *TI*^+^, and *TI*^++^ conditions was randomized for each participant, while the order of the custom-built runways was fixed. Practice trials allowed participants to become familiar with the running velocity and the desired trunk postures. Participants accomplished ten valid runs per condition in which they fully struck each force plate with a single foot, so the second force plate was always hit by the right (dominant) foot. The selected kinematic and kinetic variables were analyzed for the right limb only.

The angular displacement of the sagittal plane lower-limb joints (hip, knee, and ankle) was determined as the motion of the distal segment relative to the proximal reference. We calculated the net lower-limb joint moments by inverse dynamics using the ground reaction force, the center of pressure, a rigid linked segment model, and anthropomorphic data [25]. A vertical ground reaction force threshold of 3% body weight was used to determine the instants of foot-touchdown and toe-off [21]. The instantaneous joint powers for lower-limb joints during stance were computed as the product of joint moment and joint angular velocity. Joint moments and angular velocities were positive for leg extension. Using the trapezium method [14, 19], we integrated joint power for each lower-limb joint with respect to time over periods of positive and negative work. Then, we divided the resulting positive and negative work values by contact time to obtain average positive and negative joint powers (*P*_*hip*_, *P*_*knee*_, *P*_*ankle*_, referred to as positive and negative power from now on). Joint moments and powers were normalized to body mass. The positive (and negative) joint powers (*P*_*j*_) were summed across all lower-limb joints to yield the total positive (and negative) power *P*_*T*_ output of the limb:

$${P_{T}=P}_{hip}+P_{knee}+P_{ankle}$$ (1)

Each joint’s percentage contribution *P*_*j*,*rel*_ to total positive (and negative) power *P*_*T*_ was computed as:

$$P_{j,rel}=\left(\frac{P_{j}}{P_{T}}\right)∙100\%$$ (2)

### Data analysis and statistical analysis

For data analysis, we selected all trials completed at a controlled speed of 3.5 m s^-1^ [26] and excluded trials that deviated from the target speed by more than 5% or differed by more than 5% in velocity from step to step (calculated from the mean horizontal velocity of the L5 marker). Kinetic and kinematic data of all successful trials were analyzed using custom-written Matlab code (Mathworks Inc., MA, USA). The raw coordinate data were filtered using a fourth-order low-pass, zero-lag Butterworth filter with a 12 Hz cutoff frequency [21]. After conducting the Shapiro‐Wilk normality test, a two-way repeated measures ANOVA with post hoc Bonferroni adjustments was employed to examine the main and interaction effects of *Step* (level-step and drop-step) and *Posture* (*TI¯*, *TI*_*0*_, *TI*^+^, and *TI*^++^) on the total limb and joints negative and positive powers. Additionally, the relative contributions of joints to total limb negative and positive powers were analyzed. The statistical significance level was set at *P <* 0.05 and the data were analyzed in SPSS software (version 21.0, IBM^Ⓡ^ Co., USA). Results were expressed as mean ± SD over all participants and variables.

## Results

**Fig 1:**
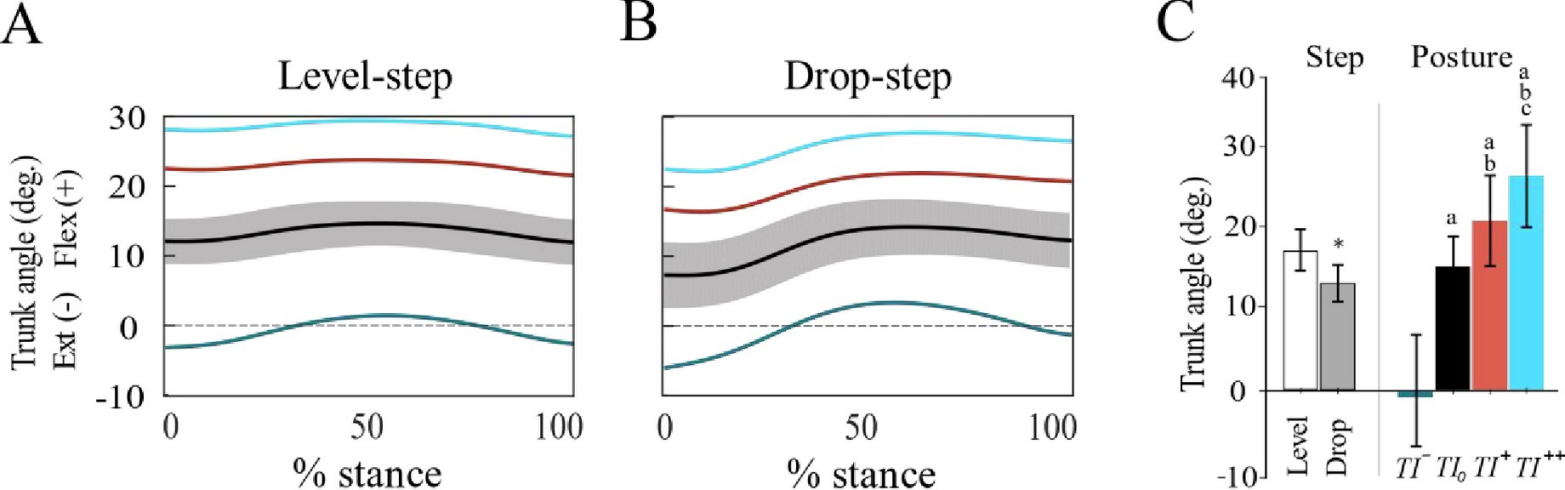
Trunk kinematics. The ensemble-averaged trunk angle across the stance phase of the level-step (A) and the drop-step (B) during running with backward (*TI¯*; green), self-selected (*TI*_*0*_; black, shaded area: ± 1 SD), low forward (*TI*^*+*^; red) and high forward (*TI*^*++*^; blue) trunk inclinations. (C) Main effects of Step and Posture on the mean trunk angle. Between-steps difference: *: significant difference from the level-step (p < 0.05). Between-postures differences: ^a^: significant difference from *TI¯*; ^b^: significant difference from *TI*_*0*_; ^c^: significant difference from *TI*^*+*^. Error bars denote ± 1 SD.

While the mean trunk angle served as an independent variable in this study, our statistical analysis aimed to demonstrate that participants were consistently capable of maintaining the target trunk inclinations. The interaction effects between *Step* and *Posture* on the mean trunk angles were not significant (*F*_1.37,15.1_ = 3.89, *P* = 0.05, *d* = 0.26); however, both *Step* (*F*_1,11_ = 3.89, *P* < 0.001, *d* = 0.84) and *Posture* (*F*_1.77,19.4_ = 105, *P* < 0.001, *d* = 0.91) had significant main effects. The mean trunk angle was significantly smaller by ~4° in the drop-step compared to the level-step and showed significant differences across all running conditions (Fig 1).

### Negative and positive powers at the hip, knee and ankle joints

**Fig 2:**
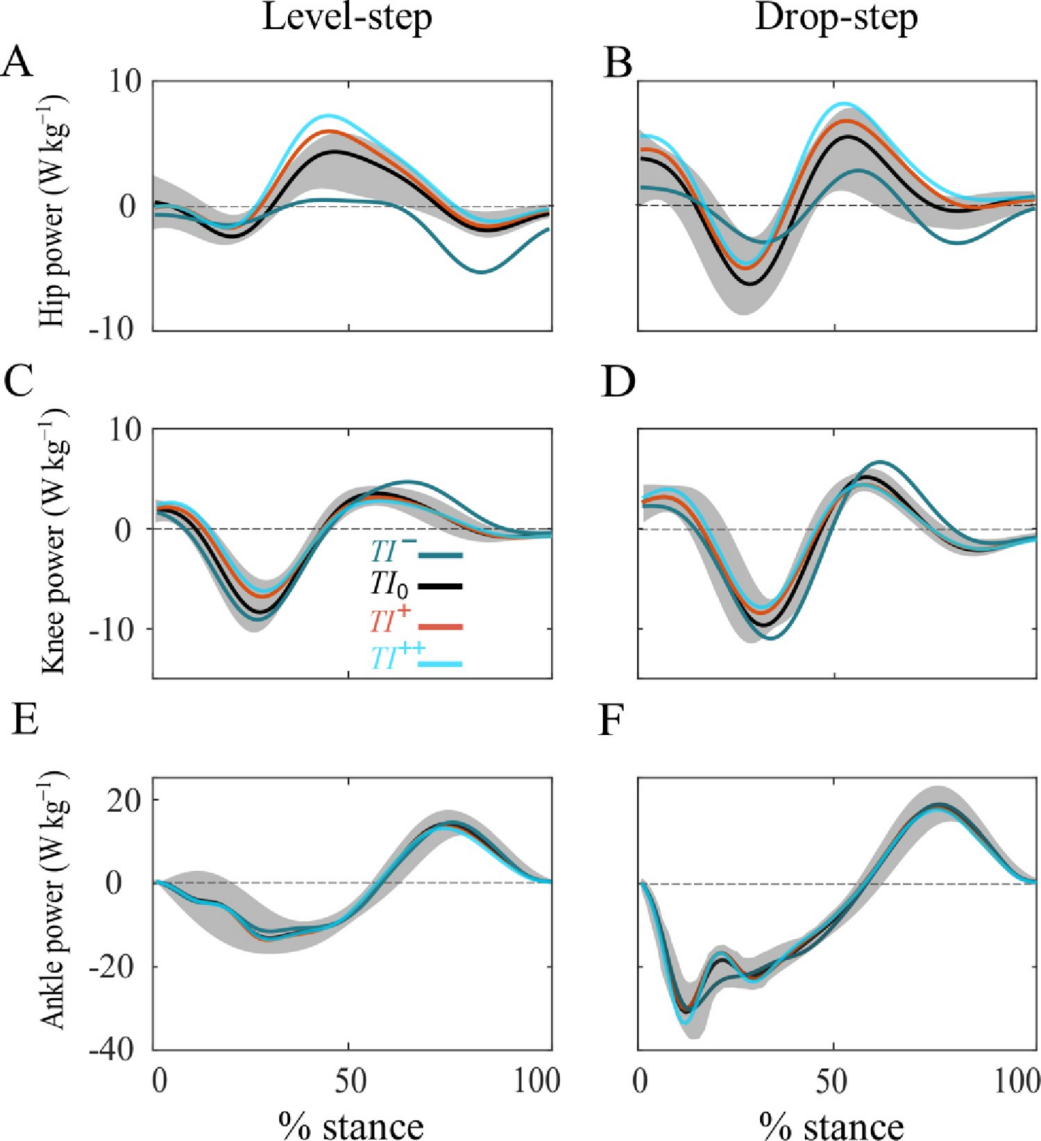
Lower-limb joints’ mechanical power. Ensemble-averaged instantaneous hip, knee and ankle powers in the level-step (left) and drop-step (right) during running with backward (*TI¯*; green), self-selected (*TI*_0_; black, shaded area ± SD), low forward (*TI*^+^; red) and high forward (*TI*^++^; blue) trunk inclinations.

**Fig 3:**
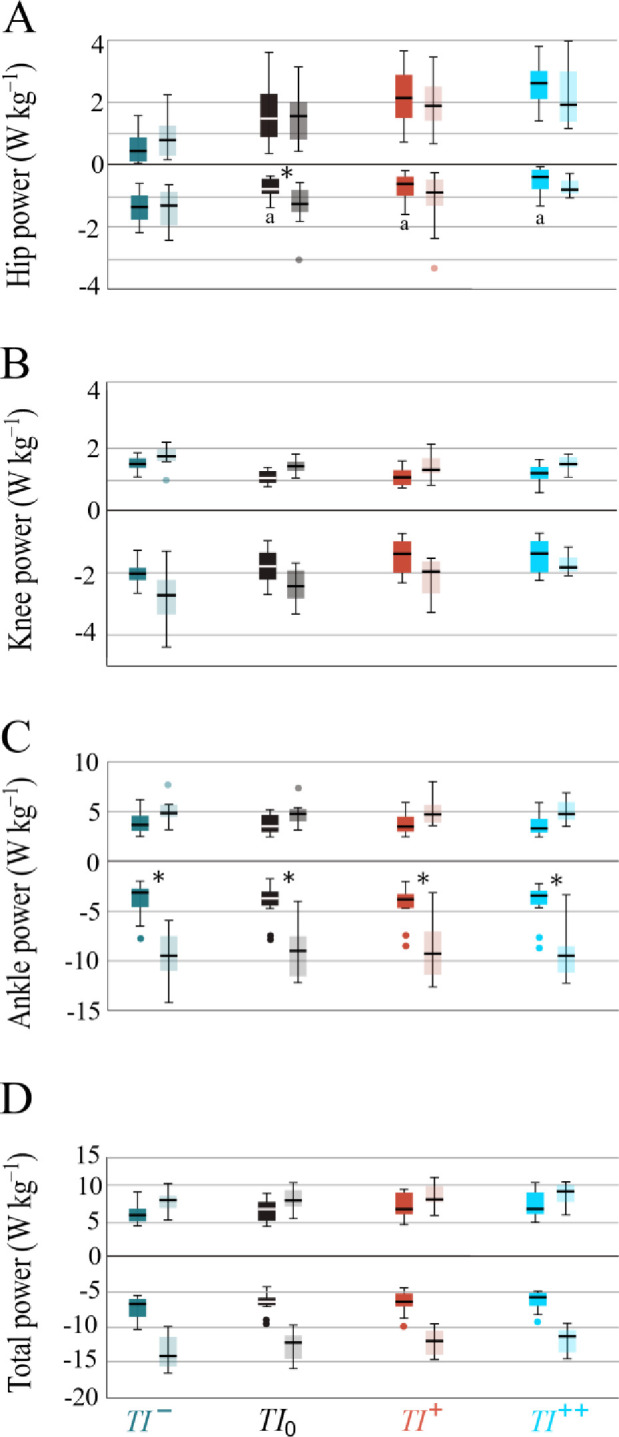
Lower-limb joints’ negative and positive powers. Boxplots depict the average negative and positive powers produced at the (A) hip, (B) knee, (C) ankle and (D) total (sum of the hip, knee and ankle) during running with backward (*TI¯*; green), self-selected (*TI*_*0*_; black), low forward (*TI*^*+*^; red) and high forward (*TI*^*++*^; blue) trunk inclinations across the level-step (darker) and the drop-step (lighter). a: significant difference from *TI¯*; *: significant difference from the level-step (p < 0.05). Error bars denote *±* 1 SD.

Overall, compared with the level-step, the magnitudes of instantaneous joint powers at the hip, knee, and ankle were subjectively greater throughout almost the entire stance phase of the drop-step for all running conditions (Fig 2). Accordingly, the average negative and positive powers increase consistently in the drop-step, with the exception of hip positive power (Fig 3).

#### Hip power

**Fig 4:**
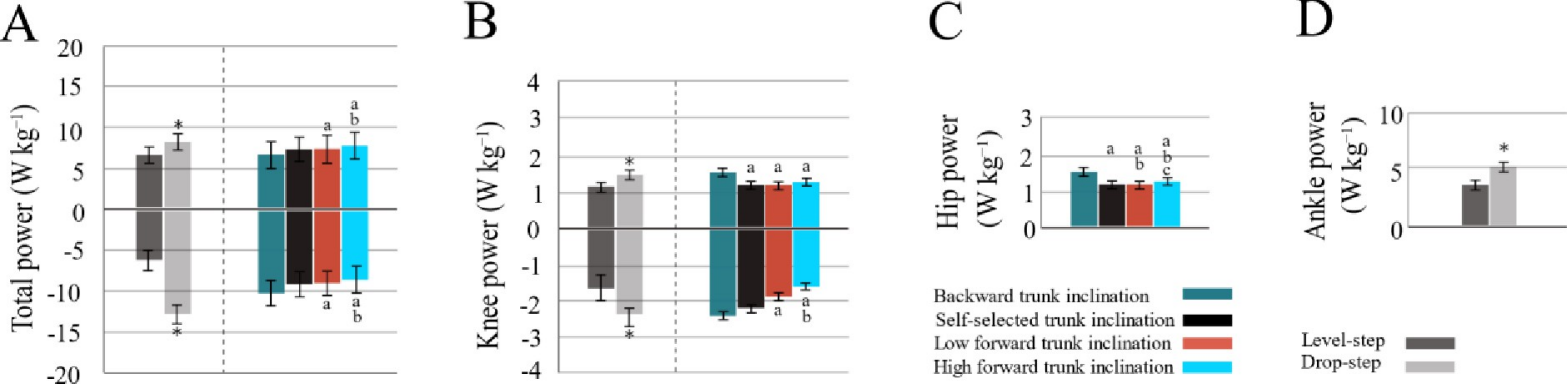
Main effects of *Step* and *Posture*. Shown are the separate main effects (mean ± SD) of *Step* (left) and *Posture* (right) for average negative and positive powers without interactions. Between-postures differences are denoted as follow: a, significant difference from *TI¯*; b, significance different from *TI*_*0*_; c, significant difference from *TI*^*+*^. Between-steps difference is indicated by * for significant difference from the level-step (p < 0.05). Error bars denote *±* 1 SD.

*Step*-by-*Posture* effects (Table 1, Fig 3A) were only found for negative hip power (*F*_3,33_ = 5.34, *P* = 0.004, *d* = 0.32). Post hoc comparisons (Fig 3A) revealed that negative hip power in the level-step during *TI¯* condition nearly doubled compared with the *TI*_*0*_ (*P* = 0.004) and *TI*^+^ (*P* = 0.002) conditions and tripled in comparison to the *TI*^++^ (*P* < 0.001) condition. The negative hip power more than doubled in the drop-step compared to the level-step, but this difference was observed only during the *TI*_*0*_ running (*P* = 0.007; Fig 3A). *Posture* had a significant main effect (*P* < 0.05) on positive hip power, showing an increase as the trunk angle transitioned from backward to forward inclination (Fig 4C).

**Table 1:** Descriptive overview of lower-limb joint average mechanical power measures.

		Posture			
	Step	*TL* ^–^	*TL*_*0*_	*TL* ^+^	*TL* ^++^
**Joint negative power (W kg** ^**˗1**^ **)**					
Hip	LS	−1.26 ± 0.53	−0.63 ± 0.33^a^	**−**0.59 ± 0.41^a^	**−**0.51 ± 0.37^a^
	DS	−1.39 ± 0.58	**−1.41 ± 0.81**	−1.12 ± 0.91	−0.85 ± 0.79
Knee	LS	−2.04 ± 0.39	−1.83 ± 0.57	−1.46 ± 0.54	−1.35 ± 0.51
	DS	−2.73 ± 0.82	−2.41 ± 0.56	−2.14 ± 0.66	−1.91 ± 0.57
Ankle	LS	−3.93 ± 1.71	−4.15 ± 1.81	−4.36 ± 1.88	−4.21 ± 1.74
	DS	**−9.47 ± 2.44**	**−8.92 ± 2.66**	**−8.89 ± 3.05**	**−9.02 ± 2.53**
Total limb	LS	−7.24 ± 1.46	−6.62 ± 1.42	−6.07 ± 1.11	−6.08 ± 1.33
	DS	−13.6 ± 2.28	−12.7 ± 2.24	−12.1 ± 1.91	−11.7 ± 1.66
**Joint positive power (W kg** ^**˗1**^ **)**					
Hip	LS	0.55 ± 0.49	1.65 ± 0.95	2.19 ± 0.91	2.59 ± 0.87
	DS	0.86 ± 0.63	1.52 ± 0.82	1.94 ± 0.85	2.37 ± 1.01
Knee	LS	1.51 ± 0.23	1.07 ± 0.22	1.08 ± 0.28	1.21 ± 0.27
	DS	1.68 ± 0.38	1.44 ± 0.22	1.41 ± 0.36	1.51 ± 0.23
Ankle	LS	3.89 ± 1.04	3.76 ± 0.92	3.76 ± 1.08	3.58 ± 1.08
	DS	5.15 ± 1.41	4.94 ± 1.34	5.01 ± 1.42	4.91 ± 1.14
Total limb	LS	5.96 ± 1.33	6.48 ± 1.53	7.05 ± 1.65	7.38 ± 1.71
	DS	7.71 ± 1.56	7.91 ± 1.61	8.38 ± 1.78	8.78 ± 1.42

Note: Data are presented as mean ± SD.

^a^ indicates significant difference from *TL*^−^across each step.

Bold values indicate significant between-step difference for each running condition.

Abbreviations: LS, level-step; DS, drop-step.

#### Knee power

Both *Step* and *Posture* had significant main effects on both negative (*Step*: *F*_1,11_ = 12.4, *P* = 0.005, *d* = 0.53; *Posture*: *F*_3,33_ = 17.6, *P* < 0.001, *d* = 0.61) and positive (*Step*: *F*_1,11_ = 14.7, *P* = 0.003, *d* = 0.57; *Posture*: *F*_3,33_ = 16.7, *P* < 0.001, *d* = 0.61) knee powers (Table 1). Post hoc comparisons (Fig 4B) revealed a significant ~37% increase in negative knee power in the drop-step compared to the level-step (*P* = 0.005). Moreover, during the *TI¯* condition, negative knee power was ~32% and ~47% greater than during the *TI*^+^ (*P* = 0.01) and *TI*^++^ (*P* = 0.006) conditions, respectively. Additionally, positive knee power exhibited ~23% increase in the drop-step compared to the level-step (*P* = 0.003) and about ~20% increase during the *TI¯* condition compared to running with other trunk inclinations (*P* < 0.01).

#### Ankle power

Significant *Step*-by-*Posture* effects (Table 1, Fig 3C) were found for negative (*F*_1.91,20.9_ = 5.46, *P* = 0.04, *d* = 0.25) but not for positive ankle power (*F*_3,33_ = 0.19, *P* = 0.91, *d* = 0.01). Negative ankle power more than doubled in the drop-step during the *TI*_*0*_ and *TI*^*+*^ conditions and nearly tripled in the *TI¯* and *TI*^*++*^ conditions compared to the level-step (*P* < 0.001; Fig 3C). No significant between-postures differences were found across the level-step (*P* **=** 0.94) and the drop-step (*P* = 0.94). Additionally, a significant *Step* main effect was observed (*F*_1,11_ **=** 50.8, *P* < 0.001, *d* = 0.82), indicating a ~30% increase in positive ankle power in the drop-step (*P* < 0.001; Fig 4D).

#### Total limb power

Both *Step* and *Posture* had significant main effects on total negative (*Step*: *F*_1,11_ = 175, *P* < 0.001, *d* = 0.94; *Posture*: *F*_3,33_ = 15.7, *P* < 0.001, *d* = 0.58) and positive (*Step*: *F*_1,11_ = 18.4, *P* = 0.001, *d* = 0.62; *Posture*: *F*_3,33_ = 13.8, *P* = 0.003, *d* = 0.55) powers (Table 1). Post hoc comparisons (Fig 4A) revealed that total negative power nearly doubled in the drop-step compared to the level-step (as indicated by the diameter of the pie graph; *P* < 0.001). It decreased in absolute value by ~8% and ~14% from *TI¯* to *TI*^*+*^ (*P* = 0.004) and *TI*^++^ (*P* = 0.002) conditions, respectively, and by ~8% from *TI*_*0*_ to *TI*^++^ (*P =* 0.005). Moreover, total positive power increased by ~22% in the drop-step compared to the level-step (*P =* 0.001) and by ~13% and ~18% from *TI¯* to *TI*^+^ (*P* = 0.02) and *TI*^++^ (*P* = 0.01) conditions, respectively.

### Relative contribution of the hip, knee and ankle to total limb power

Overall, our observation highlights the impact of *Step*-related changes in trunk inclinations during running on the relative contribution of joints to both total limb negative and positive powers.

#### Hip contribution

**Fig 5:**
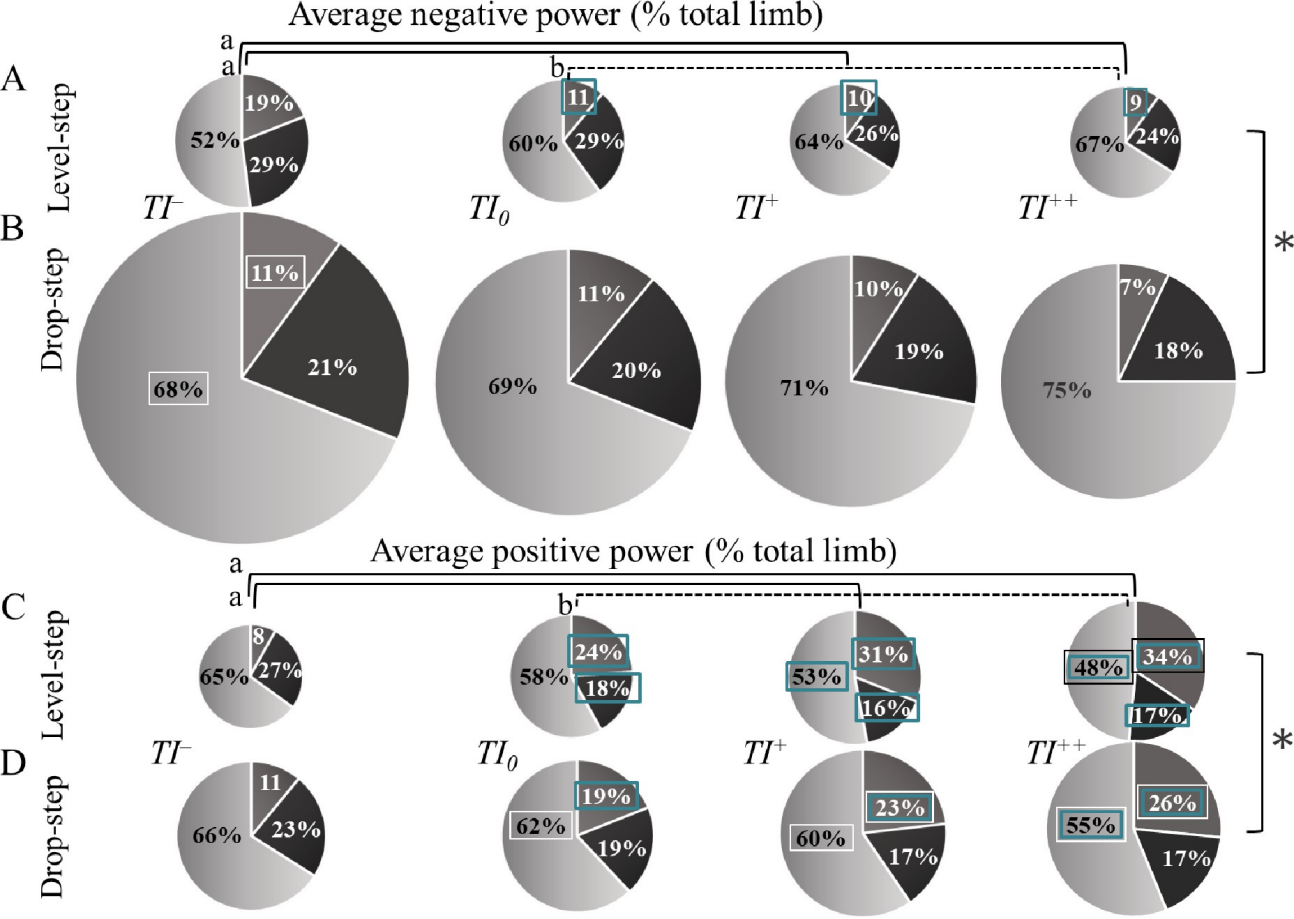
The relative contribution of the hip, knee and ankle joints to total limb power. Pie charts depict the percentage of total average negative (A, level-step; B, drop-step) and positive (C, level-step; D, drop-step) power contributed at the hip (darker grey), knee (black) and ankle (lighter grey) joints during running with backward (*TI¯*), self-selected (*TI*_*0*_), low forward (*TI*^*+*^) and high forward (*TI*^*++*^) trunk inclinations. Green and black boxes indicate significant differences from *TI¯* and *TI*_*0*_, respectively, while white boxes indicate a significant difference from the level-step. The size of each pie represents total average negative or positive power. In the absence of interaction effects, the horizontal and vertical lines denote significant differences between posture (in case of a *Posture* main effect) and step (in case of a *Step* main effect) conditions, respectively.

Significant *Step*-by-*Posture* effects were found for the hip’s relative contribution to total limb negative (*F*_3,33_ = 13.7, *P* < 0.001, *d* = 0.55) and positive (*F*_2,22_ = 8.44, *P* < 0.001, *d* = 0.43) powers. Post hoc comparisons (Table 2, Fig 5B) revealed that in the drop-step (*P* = 0.72), there were no significant between-postures differences in the relative contribution of the hip to total limb negative power. However, in the level-step during the *TI¯* condition, the relative contribution was significantly greater than during the *TI*_*0*_ (*P* = 0.02), *TI*^*+*^ (*P* = 0.02), and *TI*^*++*^ (*P =* 0.009) conditions (Fig 5A; highlighted by the green boxes). Furthermore, between-steps comparisons showed a significant decrease in the relative contribution in the drop-step during the *TI¯* condition only (*P* = 0.005; Fig 5B). The relative contribution of the hip to total limb positive power was significantly lower in both the level-step (*P* < 0.001) and the drop-step (*P* < 0.001) during *TI¯* running compared to other conditions (Fig 5C and 5D, highlighted by the green boxes). It also decreased in the drop-step compared to the level-step during the *TI*^*+*^ (*P* = 0.02) and *TI*^*++*^ (*P* = 0.02) conditions (highlighted by the white boxes).

**Table 2:** Relative contribution of each joint to total limb power.

		Posture			
	Step	*TL* ^–^	*TL*_*0*_	*TL* ^+^	*TL* ^++^
**Joint negative power (%)**					
Hip	LS	19 ± 7	11 ± 5^a^	10 ± 6^a^	9 ± 6^a^
	DS	**11 ± 4**	11 ± 7	10 ± 9	7 ± 8
Knee	LS	29 ± 8	29 ± 11	26 ± 8	24 ± 10
	DS	21 ± 7	20 ± 6	19 ± 8	18 ± 7
Ankle	LS	52 ± 12	60 ± 14	64 ± 14	67 ± 14
	DS	**68 ± 9**	69 ± 13	71 ± 17	75 ± 17
**Joint positive power (%)**					
Hip	LS	8 ± 6	24 ± 9^a^	31 ± 8^a^	34 ± 7^a^^,^^b^
	DS	10 ± 7	18 ± 8^a^	**22 ± 8** ^a^	**26 ± 9** ^a^
Knee	LS	27 ± 7	18 ± 6^a^	16 ± 5^a^	17 ± 5^a^
	DS	22 ± 6	18 ± 3	17 ± 4^a^	17 ± 3
Ankle	LS	65 ± 6	58 ± 8	53 ± 8^a^	48 ± 7^a^^,^^b^
	DS	66 ± 7	**62 ± 8**	**59 ± 8**	**55 ± 8** ^a^

Note: Data are presented as mean ± SD.

^a^ indicates significant difference from *TL*^−^across each step.

^b^ indicates significant difference from *TL*_0_ across each step.

Bold values indicate significant between-step difference for each running condition.

Abbreviations: LS, level-step; DS, drop-step.

### Knee contribution

Significant *Step*-by-*Posture* effects were found for the knee’s relative contribution to total limb positive power (*F*_3,33_ = 5.11, *P* < 0.001, *d* = 0.31). In the level-step, this relative contribution was higher during *TI¯* (*P* < 0.001) than in other conditions (Table 2, Fig 5C), while in the drop-step, it was only greater than *TI*^*+*^ condition (*P* = 0.04). No between-steps differences were detected across postures.

### Ankle contribution

Significant *Step*-by-*Posture* effects were found for the ankle’s relative contribution to both total limb negative (*F*_3,33_ = 6.12, *P* = 0.002, *d* = 0.35) and positive (*F*_3,33_ = 3.99, *P* = 0.01, *d* = 0.26) powers (Table 2). The relative contribution of the ankle to total limb negative power increased in the drop-step compared to the level-step during *TI¯* running (*P* < 0.001; Fig 5B). It also contributed significantly more to total limb positive power in both the level-step and the drop-step compared to the *TI*^+^ and *TI*^*++*^ conditions (*P <* 0.01). Additionally, the relative contribution of the ankle to total limb positive power increased in the drop-step during *TI*_0_ (*P =* 0.04), *TI*^*+*^ (*P =* 0.01), and *TI*^*++*^(*P =* 0.004) conditions (Fig 5D; highlighted by the white boxes).

## Discussion

This study aimed to characterize the posture-induced power redistribution in the lower-limb joints, along with the relative contribution from each joint towards total limb average negative and positive powers during perturbed running. The data strongly corroborate our first hypothesis that the perturbed limb (stepping into a hole; drop-step) during running would be associated with an increase in both total limb negative and positive powers (Fig 3). Likewise, data confirmed our second hypothesis that total limb negative power would decrease, and total limb positive power would increase with increasing trunk inclination. The results provided further support for our third hypothesis that stepping into a hole during running would result in a proximodistal shift in the joint contribution to total limb negative power when the trunk is more vertical, while an opposing shift in joint contributions to total limb positive power occurs with increasing trunk flexion angle.

The distribution and relative contribution of the lower-limb joints varied in response to the specific mechanical demands of locomotor behavior. A more upright trunk orientation favored energy dissipation over generation, resulting in significant changes in the individual joint power contributions to total limb power. Accommodating a discrete drop-step perturbation required an increase in both total limb negative and positive powers with an influence of trunk posture on the pattern of lower-limb joint contributions to total limb power. An upright trunk orientation led to a proximodistal shift in the relative contribution to total limb negative power. In contrast, increasing trunk flexion angle resulted in a distoproximal shift in the relative contribution to total limb positive power. Specifically, when negotiating a drop-step with an extended trunk, the hip and knee contributed approximately 50% and 30% less to total limb negative power, respectively, while the ankle’s contribution increased by about 30%.

Our study aligns with previous research, demonstrating the modulation of total limb positive and negative work or power in response to locomotion demands, with distinct roles of lower-limb joints. For instance, decelerating during level running seems to be achieved by reducing acceleration forces rather than increasing deceleration forces [27]. In incline running, the magnitude of limb negative and positive powers decreases and increases, respectively, during uphill running, while the opposite occurs when downhill running [20]. The knee emerges as the primary contributor to negative power, while the ankle remains the dominant source of positive power. Our study reveals a consistent pattern, akin to level running or uphill and downhill running [20]: the ankle serves as the primary source for both lower-limb negative (52–75%) and positive (48–66%) mechanical powers, irrespective of ground surface properties or trunk inclination angle. Additionally, in human hopping responses to unexpected perturbations, effective recovery strategies emerge in scenarios like falling into a hole. Perturbation heights, ranging from 5 to 10 cm, lead to a shift in energy absorption toward the ankle. However, with a perturbation height of 20 cm, there’s a transition to proximal lower-limb joints—the knee and hip—which absorb mechanical energy and stabilize recovery. This strategic shift reduces stress on muscles and tendons by directing mechanical power toward larger proximal muscles. Birds also display a similar pattern, with proximal joints (hip and knee) playing consistent roles in level running, while distal joints adapt for stability [28].

Comparing our findings to studies on human hopping poses challenges due to differing locomotor behaviors and perturbation characteristics (type, magnitude, and subjectivity), impacting lower-limb joint mechanics coordination. The observed increase in ankle power in our study may stem from a blend of feedforward activation, anticipating the drop-step, and subsequent proprioceptive feedback modulation during stance. The distal segments, sensitive to ground changes, exhibit high intrinsic mechanical sensitivity and proprioceptive feedback gains [28], contributing to this occurrence. This might signify an active muscle-tendon strategy by ankle plantar flexors, potentially aimed at safeguarding against muscle injuries from forceful lengthening of muscle fascicles during stepdown perturbations.

Our findings suggest posture-induced redistribution of lower-limb joints’ positive and negative powers and, consequently, the total limb mechanical power. The location of the center of mass relative to the hip in human locomotion affects lower-limb operation and energetics [23]. The anterior center of mass position with respect to the hip, resulting from a slight forward trunk lean in human habitual upright gait [29], promotes the limb’s elastic storage. Human running simulations illustrate that a posterior shift of the center of mass relative to the hip increases hip energy absorption, while the opposite shift results in positive net hip work compensating for axial losses [23]. Our experimental results mirror these mechanical changes in the hip when transitioning from a slightly backward trunk orientation to a more flexed one (Fig 3A). Additionally, joint contributions to total limb negative or positive power adapt to perturbation demands (e.g., drop-step, Fig 3A), resulting in an increased hip negative power with an extended trunk and heightened hip positive power with a more flexed trunk posture. These findings highlight the sensitivity of hip energetics to postural inclination and ground conditions.

A drop-step during running led to increased negative and positive knee power. Rapid, unanticipated changes in ground height during hopping have also shown an elevation in negative knee power [19]. The knee emerges as the primary source of negative joint work during downhill walking [13] and running [20]. Knee extensors (e.g., quadriceps) generate greater eccentric work and thus compensatory energy dissipation during the braking phase in the drop-step to control knee flexion and mitigate the vertical drop of the center of mass. Additionally, the knee generates greater positive power in the propulsion phase (Fig 5B) to propel the center of mass forward and upward back to level.

Furthermore, we observed decreased negative and positive knee powers with increasing trunk flexion, aligning with previous studies on sagittal trunk posture and lower-limb joint mechanics [3, 11]. Our findings also highlight that the knee’s relative contribution to total limb negative or positive power remains consistent with trunk posture modifications. However, its contribution to total limb positive power decreases as trunk flexion increases. This decrease is primarily due to the shift of the ground reaction force’s line of action toward the knee’s axis of rotation, resulting in smaller moment arms and decreased net external moments [2, 26]. Although negative power at the knee substantially increased when accommodating a drop-step or leaning the trunk backward, these results contrast with studies suggesting that the knee is the primary source of negative power generation [20, 30]. This discrepancy may stem from differences in the locomotor tasks, eliciting different patterns of load redistribution across lower-limb joints.

The negative power generated by the ankle more than doubled in the drop-step, irrespective of trunk orientation. This phenomenon is associated with an earlier muscle activation within the muscle-tendon unit length change cycle in the drop-step [31]. Compared to the steady cycles in level running, this results in an increased generation of negative work, allowing the muscle-tendon unit to absorb more energy and enhance energy dissipation [31]. The contribution of each lower-limb joint to the total limb power profile varies with the vertical height of the perturbation in the ground surface. For instance, when accommodating perturbation heights of ≤ 10 cm during human hopping, the energy absorption occurs mainly at the ankle, whereas for those up to 20 cm, the knee and hip joints become predominant energy absorbers [19]. A proximodistal distribution pattern in joint neuromechanical control also characterizes birds’ locomotion in response to an unexpected drop in substrate height. The mechanical role of the proximal joints (hip and knee) in negotiating changes in ground surface level remains consistent with level running, indicating load-insensitive mechanical performance. In contrast, the distal joints, namely the ankle and tarsometatarso-phalangeal joints, demonstrate a swift transition between spring-like and damping functions based on the limb’s posture at ground contact. This adaptability is crucial for maintaining stability [28].

Irrespective of ground surface properties or trunk inclination angle, our findings illustrate that the ankle consistently serves as the dominant source of negative and positive powers. This observation holds true not only during progressively increasing running speeds on flat surfaces [14] but also during uphill and downhill running [20]. Increased energy dissipation may represent a conservative strategy prioritizing safety over economy to maintain stability during a stepdown perturbation. The substantial increase in ankle power in the drop-step (Fig 5A) may be attributed to altered feedforward activation due to anticipation and subsequent proprioceptive feedback modulation. The distal segments, the first to sense ground surface changes, likely possess high intrinsic mechanical sensitivity and proprioceptive feedback gains [28].

Biomechanical responses to perturbations offer crucial insights into the stability and functionality of biological systems, holding significant implications for biomedical technology and clinical practice. Understanding how the human locomotor system adapts to postural and environmental constraints, particularly in terms of joint-level energy dynamics, is pivotal for optimizing assistive devices and rehabilitation interventions. Our study underscores the importance of considering trunk flexion in assessing lower-limb energetics during perturbed running, highlighting valuable clinical implications. For instance, we demonstrate the redistribution of lower-limb joint powers due to changes in upper-body orientation, providing critical insights for designing humanoid exoskeletons with actuated hip, knee, and ankle joints tailored to individual gait characteristics. Moreover, future research integrating joint-level mechanical analysis with the study of *in vivo* muscle-tendon behavior during anticipated or unanticipated perturbations holds promise for developing more effective rehabilitation strategies. By elucidating underlying neuromotor control strategies, these advancements can enhance the performance of rehabilitation assistive devices and interventions in addressing biomechanical deviations and minimizing injury risk in individuals with gait disturbances or lower-limb injuries.

While our study offers novel insights into the coordination of lower-limb mechanical power and joint dynamics in response to upper-body adjustments during a drop-step perturbation in running, we acknowledge several methodological limitations. Firstly, our use of inverse dynamics for estimating joint mechanics cannot differentiate between the co-contraction of synergistic and antagonistic muscles acting at each joint, as it accounts for net joint moments and powers only. Moreover, the single-joint analysis ignores effects of two-joint muscles. Recognizing that joint mechanical powers does not fully represent muscle-tendon performance, future research could incorporate *in vivo* recordings, such as dynamic imaging techniques and electromyography, to explore muscle-tendon level strategies. Secondly, our inverse dynamics analysis did not include the mechanical contribution of the elastic foot segment despite its role as a viscous spring damper element during running [32]. Future investigations should consider the foot’s role in energy dissipation in (un)perturbed paradigms and various functional tasks. Thirdly, while we did not strictly control forward and backward trunk inclinations, we observed statistically significant changes in the mean trunk angle between conditions. Lastly, participants had prior knowledge of both the postural task and the location of the drop-step perturbation, potentially influencing joint-level mechanical adjustments through feed-forward control. Future studies could enhance the ecological validity of our research outcomes by investigating joint mechanical behavior in response to unexpected perturbations.

## Conclusions

In summary, our study reveals significant alterations in the mechanical power profiles of lower-limb joints related to posture during perturbed locomotion. When encountering a hole while running, there is a consistent increase in total limb positive and negative powers, with the ankle emerging as the primary contributor to lower-limb energy absorption or generation, regardless of trunk posture. Notably, an increased forward inclination of the trunk results in a distoproximal shift in limb positive power, while an upright trunk orientation elicits the opposite effect in limb negative power. These findings shed light on the intricate interplay between posture and mechanical power distribution during perturbed locomotion.
